# Silicon Fertilizer Application Promotes Phytolith Accumulation in Rice Plants

**DOI:** 10.3389/fpls.2019.00425

**Published:** 2019-04-16

**Authors:** Xing Sun, Qin Liu, Tongtong Tang, Xiang Chen, Xia Luo

**Affiliations:** ^1^Institute of Soil Science, Chinese Academy of Sciences, Nanjing, China; ^2^School of Biological Science and Food Engineering, Chuzhou University, Chuzhou, China

**Keywords:** Si fertilizer, phytolith accumulation, Si-deficient paddy soils, PhytOC, rice organs

## Abstract

In this study, a pot experiment was designed to elucidate the effect of varying dosages of silicon (Si) fertilizer application in Si-deficient and enriched paddy soils on rice phytolith and carbon (C) bio-sequestration within phytoliths (PhytOC). The maximum Si fertilizer dosage treatment (XG3) in the Si-deficit paddy soil resulted in an increase in the rice phytolith content by 100.77% in the stem, 29.46% in the sheath and 36.84% in the leaf compared to treatment without Si fertilizer treatment (CK). However, the maximum Si fertilizer dosage treatment (WG3) in the Si -enriched soil increased the rice phytolith content by only 32.83% in the stem, 27.01% in the sheath and 32.06% in the leaf. Overall, Si fertilizer application significantly (*p* < 0.05) increased the content of the rice phytoliths in the stem, leaf and sheath in both the Si-deficient and enriched paddy soils, and the statistical results showed a positive correlation between the amount of Si fertilizer applied and the rice phytolith content, with correlation coefficients of 0.998 (*p* < 0.01) in the Si-deficient soil and 0.952 (*p* < 0.05) in the Si-enriched soil. In addition, the existence of phytoliths in the stem, leaf, and sheath of rice and its content in the Si-enriched soil were markedly higher than that in the Si-deficient soil. Therefore, Si fertilizer application helped to improve the phytolith content of the rice plant.

## Introduction

Phytoliths derive from bio-mineralization in plants and usually take the shape of the plant cell or cell spatium where Si is deposited. The phytolith content of plants ranges from less than 50 g kg^-1^ to as high as 150 g kg^-1^ (Epstein, 1994; Parr et al., 2010; Song et al., 2013, 2017; Ji et al., 2017), mainly due to phylogenetic differences in Si requirements of most dicotyledons and some Gramineae (Hodson et al., 2005), as well as the amount of available silica in the environment (Seyfferth et al., 2013; Guo et al., 2015; Si et al., 2018; Wen et al., 2018).

Rice is a staple crop, with a global planting area of approximately 1.64 × 10^8^ ha as of 2014 (Prajapati et al., 2016). When rice is harvested, the rice straw and husks are removed from the paddy field and used for other purposes, including animal feeding and firewood, or simply incinerated (Savant et al., 1996). Thus, most of the Si taken up by rice is removed from a field when the rice straw is removed, and the loss of SiO_2_ is from 75 to 130 kg hm^-2^ every production season (Zhang et al., 2014). Such large losses of Si make it difficult to maintain the balance of Si in soils from natural weathering alone. Currently, most paddy soils in China are Si-deficient. For example, 73% of paddy soils in Zhejiang Province and approximately 60% in Henan Province are Si-deficient (Cai, 2015). Some research has shown that Si fertilizer application can significantly increase the biomass of rice (Wu et al., 2014; Zhang et al., 2014).

In plants, monosilicic acid is taken up from the soil by a specific transporter (Ma et al., 2006; Song et al., 2014a) and deposited throughout the cellular structures, thereby forming amorphous Si particles known as “phytoliths” (Piperno, 1988; Pearsall, 1989). There is a significant correlation between the Si content and the phytolith content of crop materials, including the leaves, stems and sheaths, and the Si concentration of the plant phytoliths is approximately 90% (Song et al., 2014a).

Phytoliths can occlude small amounts of many elements, such as C, N, S, and so on (Kameník et al., 2013; Li et al., 2014; Anala and Nambisan, 2015). The C-occluded content of phytoliths ranges from less than 1 g kg^-1^ to as high as 100 g kg^-1^ (Clarke, 2003). This PhytOC can be stored in the soil for thousands of years (Parr and Sullivan, 2005). Thus, it plays a vital role in global carbon (C) pools (Song et al., 2014a). The Si cycle is tightly coupled to the C cycle, and this interaction is relevant for research on climate change (Chadwick et al., 1994). The formation of phytoliths in rice plants depends not only on the crops (Li et al., 2013a; Guo et al., 2015) but also on the plant cultivars (Hodson et al., 2005; Henriet et al., 2008; Yang et al., 2015), the soil’s Si availability (Henriet et al., 2008; Klotzbücher et al., 2018) and so on.

The application of Si fertilizer in soils with different available Si contents needs further study regarding the accumulation of phytoliths in rice. Thus, in this work, we designed a pot experiment to elucidate the effect of varying dosages of Si fertilizer application on the rice phytolith and PhytOC contents of plants grown in Si-deficient and enriched paddy soils.

## Materials and Methods

### Experimental Soils and Rice Cultivar

The Si-deficient paddy soil (red paddy soil) was obtained from Yangliu Town, Xuanchen City, Anhui Province, China. The Si-enriched paddy soil (Wushan soils) was obtained from the Changshu Agroecological Experimental Station, Chinese Academy of Sciences. The base is located in Xinzhuang County, South Changshu, Suzhou, Jiangsu Province, China. The physicochemical properties of the two soils are shown in Table 1.

**Table 1:** Basic chemical properties of the two soils.

Experimental Soils	NH_4_OAc- extractable Si (mg kg^-1^)	pH	Total N (g kg^-1^)	Total P (g kg^-1^)	Total K (g kg^-1^)	Organic matter (mg kg^-1^)	Na_2_CO_3_- extractable P (mg kg^-1^)	NH_4_OAc- extractable K (mg kg^-1^)
Si-deficient paddy soil (red paddy soil)	5.67	4.62	1.20	0.18	52.49	28.89	17.44	210.0
Si-enriched paddy soil (Wushan soils)	252.3	7.54	2.40	0.73	20.16	39.89	34.27	101.7


The rice cultivar (*Oryza* sativa) Nanjing 46 was obtained from the Changshu Agroecological Experimental Station, Chinese Academy of Sciences.

### Pot Experiment

Two soils (Si-deficient and enriched paddy soils) were selected from Xuanchen City and the Changshu Agroecological Experimental Station, Chinese Academy of Sciences, respectively. Four available Si dosages were designed in the pot experiments: (1) CK (Si fertilizer not applied); (2) low slag Si fertilizer I (SiO_2_150 kg ha^-1^); (3) high slag Si fertilizer II (300 kg ha^-1^); and (4) high slag Si fertilizer III (600 kg ha^-1^). Thus, this experiment comprised 8 treatments repeated 3 times. Two soils were placed in the pot bowl for a total volume of 0.0175 m^3^; each pot contained N 46%, P_2_O_5_ 13.5%, and K_2_O 60%, Si fertilizer was applied as the base fertilizer and three rice plants were planted in every pot. Pots were placed in the greenhouse of the Changshu Agroecological Experimental Station, Chinese Academy of Sciences in June 2014, and the whole rice growth period was maintained using conventional management.

### Sample Preparation

After the rice cultivar harvest, each rice plant was separated into five different organs: sheath, leaf, root, stem, and grain. All rice samples were rinsed twice in distilled water, placed in an ultrasonic bath for 20 min and subsequently dried in oven at 70°C for 24 h. After hulling, the rice organ samples were stored for phytolith extraction and PhytOC determination.

### Phytolith Extraction From Rice Organs and PhytOC Analysis

**FIGURE 1:**
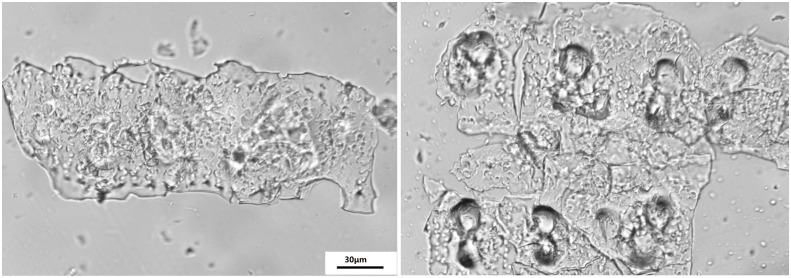
Optical microscope images of phytoliths extracted from the rice samples using the wet ashing method according to Zuo and Lü (2011) and Sun et al. (2016); magnification 400×, scale bar 30 μm.

The phytolith extraction was used for a revised wet digestion measurement previously described by Zuo and Lü (2011); Sun et al. (2016). Phytolith extraction sample assemblages were installed on glass slides in Balsam Canada mounting medium. The slides were viewed at 400 × magnification using a microscope (Jiangnan XP-213, China) fitted with a polarizing filter and a 5.0 MP color CCD camera to ensure the absence of organic material residue as shown by Parr et al. (2010; Figure 1). The PhytOC was measured using an Elemental Analyzer 3000 (GmbH Company, Germany).

### Statistical Analyses

The mean values of all parameters were calculated from the determination of three replicates, and the standard errors of the means were determined. A one-way ANOVA was used to measure the significance of the results between different varieties, and Tukey’s multiple range tests (*p* < 0.05) were subsequently performed. All the statistical analyses were performed using SPSS v.17 for Windows.

## Results

### Phytolith and C Contents of the Phytoliths in the Rice Organs

With an increase in the application of the Si fertilizer dosages, the content of the phytoliths in the rice organs was increased in the Si-deficient red paddy soil (Table 2). For example, the content of the phytoliths in the XG3 (26.10 g kg^-1^) and XG2 (18.50 g kg^-1^) stems was significantly (*p* < 0.05) higher than that of the control (13.00 g kg^-1^), and the rate increased by 100.7 and 42.3%, respectively. In addition, the content of the phytoliths of XG1 in the stem was not significantly (*p* > 0.05) different than that of the control. However, the content of the phytoliths in the rice sheath and leaf could be significantly (*p* < 0.05) increased by the application of all the Si fertilizer dosages. The content of the phytoliths in the XG3 treatment rice grains could only be increased by a high dose of Si fertilizer application. However, the content of the phytoliths in all the root treatments was not significantly (*p* > 0.05) different from that of the control.

**Table 2:** Different effects of silicon fertilizers on rice organs content of phytoliths.

Treatments	stem (g kg^-1^)	sheath (g kg^-1^)	leaf (g kg^-1^)	grain (g kg^-1^)	root (g kg^-1^)
XCK	13.00 ± 1.44c	36.01 ± 1.45c	34.72 ± 1.50c	11.59 ± 2.41b	80.66 ± 25.81a
XG1	16.84 ± 1.07bc	42.73 ± 2.74b	41.37 ± 2.25b	13.86 ± 1.61b	117.20 ± 18.58a
XG2	18.50 ± 0.91b	49.37 ± 0.89a	48.72 ± 2.09a	12.96 ± 3.49b	92.87 ± 36.86a
XG3	26.10 ± 4.41a	46.62 ± 2.38a	47.51 ± 3.08a	19.37 ± 1.38a	84.78 ± 30.35a
WCK	37.22 ± 4.19b	75.31 ± 4.68bc	76.18 ± 4.44b	16.60 ± 3.29b	67.46 ± 22.70b
WG1	38.57 ± 4.63b	74.18 ± 3.21c	76.14 ± 8.10b	17.26 ± 1.92b	56.09 ± 1.76b
WG2	42.09 ± 3.23b	84.00 ± 5.19b	93.13 ± 11.68a	21.69 ± 1.83ab	96.66 ± 14.10a
WG3	49.44 ± 0.74a	95.72 ± 5.59a	100.60 ± 7.98a	24.72 ± 4.96a	82.03 ± 5.32ab


*W, Wushan soil; X, Red paddy soil; G, Leg silicon fertilizer. Different lowercase letters after the data mean that the difference between different types of Si-fertilizer dosage treatments are significant (*p* < 0.05)*.

With the increase in the application of the Si fertilizer doses, the content of the phytolith in the rice organs could be increased in the Si-enriched Wushan paddy soil (Table 2). For example, the content of the phytoliths of the WG3 (100.60 g kg^-1^) and WG2 (93.13 g kg^-1^) leaves was significantly (*p* < 0.05) higher than that of the control (76.18 g kg^-1^), and the increase in the rate was 32.06 and 22.25%, respectively. In addition, the content of the phytoliths of WG1 in the stem was not significantly (*p* > 0.05) different from that of the control. However, the content of the phytoliths in the other rice organs could be significantly (*p* < 0.05) increased by the application of high Si fertilizer dosages.

Thus, different Si fertilizer doses might increase the content of the phytoliths in the rice organs in either Si-deficient red paddy soil or Si-enriched Wushan paddy soil. The C content in the phytoliths in the organs was not affected by the increase in the Si fertilizer dose. However, the content of the C in the phytoliths was different in all the organs. Generally the content of the C of the leaf phytoliths was higher than that of the other organs (Table 3).

**Table 3:** Different effects of silicon fertilizers on rice organs content of C content of phytoliths.

Treatments	stem (g kg^-1^)	sheath (g kg^-1^)	leaf (g kg^-1^)	grain (g kg^-1^)	root (g kg^-1^)
XCK	8.60 ± 1.34a	5.80 ± 0.75a	8.02 ± 1.81a	4.78 ± 0.61a	1.67 ± 0.01a
XG1	4.85 ± 1.20b	6.09 ± 0.92a	9.21 ± 3.84a	6.79 ± 1.87a	1.88 ± 0.31a
XG2	5.28 ± 1.20b	4.82 ± 1.20a	9.47 ± 1.75a	7.08 ± 1.43a	1.68 ± 0.24a
XG3	4.64 ± 0.38b	4.61 ± 0.97a	7.23 ± 1.20a	5.10 ± 1.90a	2.19 ± 0.34a
WCK	2.28 ± 0.36a	2.28 ± 0.39ab	7.41 ± 2.81a	6.53 ± 2.91a	4.98 ± 1.42a
WG1	2.59 ± 0.55a	2.16 ± 0.11ab	3.22 ± 0.82a	3.39 ± 0.45a	4.79 ± 2.22a
WG2	4.13 ± 2.0a	2.82 ± 0.54a	5.58 ± 3.37a	4.34 ± 0.92a	4.68 ± 0.59a
WG3	2.23 ± 0.67a	1.90 ± 0.09b	4.31 ± 2.57a	4.42 ± 1.18a	4.03 ± 1.12a


*W, Wushan soil; X, Red paddy soil; G, Leg silicon fertilizer. Different lowercase letters after the data mean that the difference between different types of Si-fertilizer dosage treatments is significant (*p* < 0.05)*.

### Phytolith Content and the Estimated PhytOC Fluxes in Whole Rice Plants

Compared with the control treatment, the content of phytoliths in the whole rice plant was significantly (*p* < 0.05) increased by the use of a high Si fertilizer dose in the two types of soils (Table 4). The C content of the phytoliths and the PhytOC content of the dry organ weights were not significantly (*p* > 0.05) different in the rice plant. In Si-deficient red paddy soil, the estimated PhytOC fluxes were calculated by the content and proportion of the phytoliths and the C content of the phytoliths in each part of the rice plant. The results showed that the application of Si fertilizer could significantly (*p* < 0.05) increase the content of the estimated PhytOC fluxes in the whole plant with the increase in the Si fertilizer dosage. The estimated PhytOC fluxes of the XG2 (11.36 kg-CO_2_ ha^-1^ year^-1^) and XG3 (12.93 kg-CO_2_ ha^-1^ year^-1^) treatments were 43.04 and 49.70%, respectively, and were significantly (*p* < 0.05) higher than those of the control treatment (8.41 kg-CO_2_ ha^-1^ year^-1^). In the Si-enriched soil, the phytolith content of all the Si fertilizer treatments in the rice plants was higher than that of the control treatment, but it was not significantly (*p* > 0.05) different in all the Si fertilizer treatments compared with the control treatment. The estimated PhytOC fluxes of WG1 were 1.3% lower than those of the control.

**Table 4:** Different effects of silicon fertilizers on rice plant content of phytoliths, C content of phytoliths, PhytOC content of dry organ weight, and the estimated PhytOC fluxes per ha in kg of CO_2_ equivalents (kg∼e∼ CO_2_) for rice.

Treatments	Phytolith content (g kg^-1^)	C content of phytoliths (g kg^-1^)	PhytOC content of dry organs weight (g kg^-1^)	Estimated PhytOC fluxes (kg-CO_2_ ha^-1^yr^-1^)	Biomass (t ha^-1^)
XCK	24.23 ± 0.39b	5.74 ± 0.52a	0.13 ± 0.01b	8.41 ± 0.99b	16.38 ± 2.34b
XG1	26.64 ± 1.02a	6.61 ± 1.48a	0.16 ± 0.04a	10.38 ± 2.43a	18.43 ± 1.74a
XG2	28.32 ± 2.06a	6.56 ± 1.04a	0.17 ± 0.03a	11.36 ± 2.34a	18.90 ± 1.67a
XG3	31.94 ± 2.18a	5.14 ± 1.05a	0.16 ± 0.03a	12.93 ± 0.54a	20.33 ± 0.77a
WCK	34.96 ± 0.88b	5.44 ± 1.80a	0.17 ± 0.05a	8.74 ± 0.29a	17.94 ± 0.46a
WG1	36.22 ± 1.06b	3.21 ± 0.15a	0.11 ± 0.01a	7.92 ± 0.95a	17.42 ± 1.61a
WG2	48.06 ± 4,34a	4.29 ± 1.09a	0.21 ± 0.08a	11.11 ± 1.93a	17.78 ± 4.38a
WG3	53.85 ± 0.79a	4.39 ± 0.68a	0.21 ± 0.07a	9.23 ± 0.09a	14.76 ± 1.78b


*W, Wushan soil; X, Red paddy soil; G, Leg silicon fertilizer. Different lowercase letters after the data mean that the difference between different types of Si-fertilizer dosage treatments are significant (*p* < 0.05)*.

### The Correlation Coefficients Between the Six Variables of the Red Paddy Soil

As shown in Table 5, the coefficient of variation in the different factors in the Si-deficient red paddy soils was high, illustrating considerable variation among these different Si fertilizer dosages. The results demonstrated that there was a significant correlation (*R* = 0.998 and *p* < 0.01) between the phytolith content and the Si fertilizer dose. The C contents of the phytoliths were not correlated (*R* = -0.177 and *p* > 0.05) with the phytolith content in the rice plants treated with different fertilizer doses. The correlation coefficient was 0.986, indicating a significant relationship (*p* < 0.05) between the phytolith content and the estimated PhytOC fluxes. The biomass of the rice was significantly related to the phytolith content (*R* = 0.972 and *p* < 0.05) and the estimated PhytOC fluxes (*R* = 0.994 and *p* < 0.01).

**Table 5:** The correlation coefficients between the six variables of the red paddy soil.

Variables	Silicon fertilizer	Phytolith content	C content of phytoliths	PhytOC content of dry organs weight	Estimated PhytOC fluxes	Biomass
Silicon fertilizer	1					
Phytolith content	0.998**	1				
C content of phytoliths	-0.238	-0.177	1			
PhytOC content of dry organs weight	0.620	0.665	0.612	1		
Estimated PhytOC fluxes	0.973*	0.986*	-0.008	0.795	1	
Biomass	0.953*	0.972*	0.041	0.799	0.994**	1


*^∗^Correlation is significant at the 0.05 level (2-tailed). ^∗∗^Correlation is significant at the 0.01 level (2-tailed)*.

### The Correlation Coefficients Between the Six Variables of the Wushan Soil

As shown in Table 6, the coefficient of variation in the different factors in the Si-deficient red paddy soils was high, illustrating considerable variation among the different Si fertilizer doses. The results demonstrated that there was a significant correlation (*R* = 0.952 and *p* < 0.05) between the phytolith content and the Si fertilizer dose. The C contents of the phytoliths were not correlated (*R* = -0.035 and *p* > 0.05) with the phytolith content in the rice plants of different fertilizer treatments. The correlation coefficient was 0.598 and there was significant correlation (*p* > 0.05) between the phytolith content and the estimated PhytOC fluxes. The biomass of the rice was significantly correlated with the phytolith content (*R* = -0.890 and *p* > 0.05) and the estimated PhytOC fluxes (*R* = 0.076 and *p* > 0.05).

**Table 6:** The correlation coefficients between the six variables of the Wushan soil.

Variables	Silicon fertilizer	Phytolith content	C content of phytoliths	PhytOC content of dry organs weight	Estimated PhytOC fluxes	Biomass
Silicon fertilizer	1					
Phytolith content	0.952*	1				
C content of phytoliths	-0.209	-0.035	1			
PhytOC content of dry organs weight	0.599	0.796	0.526	1		
Estimated PhytOC fluxes	0.333	0.598	0.229	0.800	1	
Biomass	-0.890	-0.746	0.100	-0.393	0.076	1


*^∗^Correlation is significant at the 0.05 level (2-tailed). ^∗∗^Correlation is significant at the 0.01 level (2-tailed)*.

## Discussion

Rice accumulates Si (Seyfferth et al., 2013), and the Si concentration is approximately 10–15% in the rice plant (Marschner, 1995), with approximately 90% of the Si present in the phytolith (Wang, 1998). There was a significant correlation between the Si content and the phytolith content of the crop materials, such as the phytolith contents of the rice leaves, stems and sheaths (Song et al., 2014a). The shape of the phytoliths in the different rice organs varied (e.g., double-peaked, bulliform, and parallel dumbbell phytoliths) (Prajapati et al., 2016). Prajapati et al. (2016) reported that the phytolith content in the different rice organs (stem, sheath, leaf, and grain) ranged from 0.14 to 26.4 g kg^-1^. Similar results and trends were reported by other researchers (Li et al., 2013c; Guo et al., 2015). Our results showed that whether the paddy soil was Si-deficient or Si-enriched, the utilization of Si fertilizer could significantly (*p* < 0.05) improve the phytolith content of the rice organs (Tables 2, 3) such as the stem, sheath, leaf, grain and root. According to the formation mechanism of phytoliths, the available Si in the soil is taken up by rice plants at the roots, usually taking the shape of the plant cell or cell spatium where Si is deposited (Piperno, 1988; Ma, 2003; Neumann, 2003; Song et al., 2016). Thus, the use of Si fertilizer increased the content of effective Si in the soil (Ma et al., 2004; Liu et al., 2006; Cai, 2015) and increased the absorption capacity of Si in the rice (Li et al., 2013c; Seyfferth et al., 2013; Guo et al., 2015; Zuo et al., 2016; Huan et al., 2018), thereby increasing the phytolith content of the rice plant (Table 4).

A substantial amount of research reported that the factors of the PhytOC content were as follows: different varieties (Parr et al., 2009, 2010; Parr and Sullivan, 2011; Li et al., 2013b; Song et al., 2017; Sun et al., 2017), pest and disease resistances (Ma et al., 2002), nitrogen utilization (Zhao et al., 2016), basalt powder (Guo et al., 2015), soil-effective Si content (Song et al., 2014b; Klotzbücher et al., 2018), and net production on the ground (Blecker et al., 2006). It has been shown that Si is an important element for rice growth and the deficiency of plant-available Si may exert an adverse effect on the rice yield through biotic stresses, disease and pests, etc. (Ma, 2004; Ma et al., 2004). Our results also showed that the contents of phytolith in rice plants were different in Si-deficient and Si-enriched paddy soil. The content of Phytolith in rice plants with Si-enriched paddy soils was higher than that in rice plants with Si-deficient paddy soil (Tables 2, 4). Moreover, whether in Si-deficient or in Si-enriched paddy soils, there was a positive correlation (*p* < 0.05) between the phytolith content of rice plants and the Si fertilizer dosages (Tables 5, 6). Previous studies have demonstrated that the content of the Si (phytoliths) in crops may be promoted through Si fertilizer application (Alvarez and Datnoff, 2001; Liang et al., 2010; Mecfel et al., 2010). Further, in the Si-deficient paddy soil, the estimated PhytOC fluxes were significantly related to the Si fertilizers (*R* = 0.973 and *p* < 0.05), the phytolith content (*R* = 0.986 and *p* < 0.05) and the biomass of the rice (*R* = 0.994 and *p* < 0.01) (Table 5). However, in the Si-enriched paddy soil, the estimated PhytOC fluxes were not correlated (*P* > 0.05) with these factors. Zhang et al. (2014) showed that the yield of rice was increased 14.5% by the use of 225 kg ha^-1^ Si fertilizer; when the application of Si fertilizer was increased to 375 kg ha^-1^, the yield of the rice increased only by 10.1%. Similarly, Wu et al. also recommended the use of 225 kg ha^-1^ Si fertilizer as the most economical measure (Wu et al., 2014). We also obtained the same results. The application of Si fertilizer to the Si-enriched paddy soil did not increase the biomass of the rice but reduced it. In particular, when the amount of the Si fertilizer reached 600 kg ha^-1^, the rice biomass decreased significantly by 29.10% compared with the control treatment (Table 4). Therefore, excessive Si fertilizer not only has no benefit to the accumulation of estimated PhytOC fluxes in rice plant, but also reduces the yield of rice. However, for Si-deficient soils, the application of Si fertilizer can not only increase rice yield, but also increase the phytolith content of rice plants and the estimated PhytOC fluxes (Table 4). Thus, different Si fertilizer doses were one of the measures to improve the phytolith content and the biomass of the rice plant. Thus, how to promote the phytolith content and C content of phytoliths will require further in-depth study.

The global rice cultivation area was approximately 1.64 × 10^8^ ha in 2014 (Prajapati et al., 2016); when rice is harvested, the rice straw and husks are removed from the paddy field and used for other purposes, including animal feeding and firewood, or simply incinerated (Savant et al., 1996). Thus, most of the Si taken up by rice is removed from a field when the rice straw is removed, and the loss of SiO_2_ is from 75 to 130 kg ha^-1^ every production season (Zhang et al., 2014). Such large losses of Si make it difficult to maintain the balance of Si in soils from natural weathering alone. Appropriate dosages of Si fertilizer could solve the problem of Si deficiency in soil, and increase the biomass of rice and the content of phytolith in rice plants, and indeed result in the occlusion of increased CO_2_ in the rice plants (Liang et al., 2010; Mecfel et al., 2010). The estimated PhytOC fluxes increased from 0.49 to 4.52 Kg-e-CO_2_ ha^-1^ year^-1^ (Table 4). More than 8.04 × 10^4^ to 7.41 × 10^5^ Mg-e-CO_2_ would have been occluded within the phytolith of the rice plants per year globally. Taking the largest estimated PhytOC flux (12.93 Kg-e-CO_2_ ha^-1^ year^-1^) of the rice plants, 2.12 × 10^6^ Mg-e-CO_2_, would have been occluded within the phytolith of rice plants every year. However, the annual CO_2_ bio-sequestration within the rice phytoliths of the unit area is likely to be lower than that of other plants, such as bamboo leaf litter (1.56 × 10^7^ Mg-e-CO_2_ year^-1^) (Parr, 2006), wetland plants (4.39 × 10^7^ Mg-e-CO_2_ year^-1^) (Guo et al., 2015), grasslands (4.14 × 10^7^ Mg-e-CO_2_ year^-1^) (Song et al., 2012), millet (2.37 × 10^6^ Mg-e-CO_2_ year^-1^) (Pan et al., 2017) and sugarcane leaf (0.72 × 10^7^ Mg-e-CO_2_ year^-1^) (Parr et al., 2009). In this study, we showed that Si fertilizer application could promote the phytolith content and biomass of rice plants and further improve the estimated PhytOC flux of rice plants. Thus, the measure provided a theoretical basis for the bio-carbon sequestration of the rice plant and laid a foundation for PhytOC fixation in paddy soil by the return of straw.

## Conclusion

The use of Si fertilizer could significantly increase the phytolith content of rice plants in Si-deficient red paddy soil or Si-enriched Wushan soil. The phytolith content of rice plants was positive correlation with the Si fertilizer dose in two types paddy soil. The estimated PhytOC fluxes in Si-deficient red paddy soil had a positive correlation with the phytolith content, the biomass of the rice and the Si fertilizer dose. In this study, we estimated that the PhytOC fluxes increased from 0.49 to 4.52 Kg-e-CO_2_ ha^-1^ year^-1^. More than 8.04 × 10^4^ to 7.41 × 10^5^ Mg-e-CO_2_ would have been occluded within the phytoliths of the rice plants per year globally. Therefore, Si fertilizer application might provide a new approach to increase the atmospheric CO_2_ occluded within the phytoliths, offering a potential method.

## Author Contributions

All authors listed have made a substantial, direct, and intellectual contribution to the work. XS completed the experiments independently, carried out the data analysis, and finished the final writing of the article. QL made great contributions to guide the process of experiments. TT, XC, and XL helped in sampling, experimentation, and essay writing.

## Conflict of Interest Statement

The authors declare that the research was conducted in the absence of any commercial or financial relationships that could be construed as a potential conflict of interest.
